# Is Peracetic Acid Fumigation Effective in Public Transportation?

**DOI:** 10.3390/ijerph19052526

**Published:** 2022-02-22

**Authors:** Ewelina Kruszewska, Piotr Czupryna, Sławomir Pancewicz, Diana Martonik, Anna Bukłaha, Anna Moniuszko-Malinowska

**Affiliations:** 1 Department of Infectious Diseases and Neuroinfections, Medical University of Białystok, Żurawia 14, 15-540 Białystok, Poland; avalon-5@wp.pl (P.C.); spancewicz@interia.pl (S.P.); annamoniuszko@op.pl (A.M.-M.); 2 Department of Infectious Diseases and Hepatology, Medical University of Białystok, Żurawia 14, 15-540 Białystok, Poland; di.martonik@gmail.com; 3 Department of Microbiological Diagnostics and Infectious Immunology, Medical University of Białystok, Waszyngtona 15A, 15-269 Białystok, Poland; anna.buklaha@umb.edu.pl

**Keywords:** disinfection, peracetic acid, fumigation, fogging, public transportation

## Abstract

The COVID-19 pandemic made more people aware of the danger of viruses and bacteria, which is why disinfection began to be used more and more often. Epidemiological safety must be ensured not only in gathering places, but also in home and work environments. It is especially challenging in public transportation, which is a perfect environment for the spread of infectious disease. Therefore, the aim of the study was the identification of bacteria in crowded places and the evaluation of the effect of fumigation with peracetic acid (PAA) in public transportation. Inactivation of microorganisms in buses and long-distance coaches was carried out using an automatic commercial fogging device filled with a solution of peracetic acid stabilized with acetic acid (AA) and hydrogen peroxide (H_2_O_2_). Before and after disinfection, samples were taken for microbiological tests. The most prevalent bacteria were *Micrococcus luteus* and *Bacillus licheniformis.* *Staphylococcus epidermidis* was only present in buses, whereas *Staphylococcus hominis* and *Exiguobacterium acetylicum* were only present in coaches. Statistical analysis showed a significant reduction in the number of microorganisms in samples taken from different surfaces after disinfection in vehicles. The overall effectiveness of disinfection was 81.7% in buses and 66.5% in coaches. Dry fog fumigation with peracetic acid is an effective method of disinfecting public transport vehicles.

## 1. Introduction

In times of the global COVID-19 pandemic, maintaining the cleanliness and hygiene of closed rooms in which we are staying is a significant problem. COVID-19 can be spread through the respiratory droplets of an infected person or through direct contact with contaminated objects or surfaces. The pandemic made more people aware of the danger of viruses and bacteria, therefore various countries have introduced non-pharmaceutical interventions (NPIs), such as the increasing use of disinfection, wearing masks and social distancing [1,2]. In the United States, Chang et al. compared the number of disinfectant poisoning exposures reported during the pandemic and during the same pre-pandemic period. They showed an overall increase of such notifications by 20.4% compared to 2019 and by 16.4% over the same period in 2018 [3]. Gharpure et al. conducted an online survey in which a third of respondents used recommended high-risk practices related to the prevention of SARS-CoV-2 virus transmission, such as cleaning their food with bleach, or even consuming cleaning agents and disinfectants [4]. Epidemiological safety must be ensured not only in gathering places, but also in home and work environments. This is especially challenging in public transportation, as it is confined space conducive to the transmission of infectious agents. A key element of everyday life is cleaning and disinfection of frequently used surfaces and rooms [5,6,7,8]. The bacteria commonly prevalent in an the environment and found in public transport are detailed in Table 1 [9,10,11,12,13,14,15,16,17,18,19].

Chemical factors that can be distinguished in a wide range of disinfectants are peracetic acid (PAA), hydrogen peroxide (H_2_O_2_) and chlorine [20,21,22,23,24,25,26,27]. Otterspoor and Farrell have proven the advantage of using peracetic acid-based disinfectants. The use of disinfectants containing hydrogen peroxide and chlorine resulted in several adverse reactions in medical personnel and more frequent reports of incidents related to work safety [22]. PAA is effective and safe for the environment, which is why it is so willingly used in medical care centers and in the food processing industry [28,29]. Studies conducted by Leggett et al. confirmed synergistic effect of the combination of PAA and H_2_O_2_ on biocidal activity, which increases the inactivation efficacy of the individual chemical factors. The synergistic effect is likely based on the weakening of the spore defensive barrier by hydrogen peroxide allowing for better penetration of peracetic acid and resulting in the enhanced sporicidal activity of the solution [30].

There are many methods of disinfection, including fumigation. The dry fog system produces submicron disinfectant droplets which evenly cover all surfaces in closed spaces without wetting them. The chemical sprayed this way has a broad spectrum of activity against pathogens, including spores [31,32].

The sporicidal oxidant PAA is widely used in the disinfection of various surfaces in the medical field [33,34]. We have examined the use of peracetic acid in schools and kindergartens, where the high effectiveness of this type of disinfection in educational institutions has been proven [35]. Considering the positive outcomes, we decided to expand the scope of our research and verify the effect of using peracetic acid in the disinfection process in buses and coaches. Another aim was to determine types of bacteria we are exposed to when using public transport.

## 2. Materials and Methods

### 2.1. Selection of Sampling Sites

**Figure 1:**
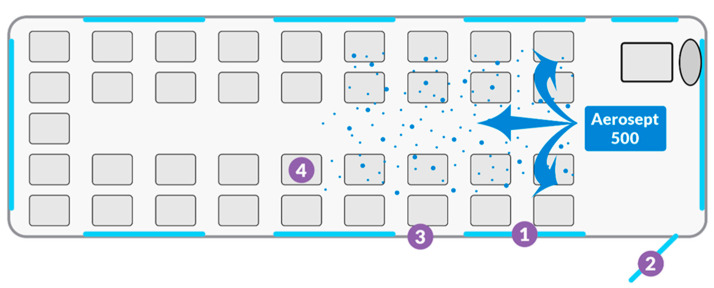
Sample picture of vehicle disinfection and sampling from individual surfaces (1—windows, 2—doors, 3—walls, 4—seatbacks).

Nine public transport buses in Białystok, Poland and nine long-distance coaches running between cities in Poland were selected at random for the experiment. Samples were taken from 4 different surfaces of each vehicle before and after disinfection. Tested surfaces i.e., windows (1), doors (2), walls (3) and the seat backs (4) are shown in Figure 1. A total of 144 prints were collected for microbiological testing.

### 2.2. Preparation and Disinfection Process

Prior to the fogging process, all openings such as windows and doors were closed and then sealed with plastic films and adhesive tapes.

The sampling was performed with a dedicated apparatus (BIOMAXIMA, Lublin, Poland) with a constant pressure of 500 g for a constant time of 10 s to ensure reproducible collection of each sample. For microbiological testing, samples from four surfaces of 18 vehicles were collected using aforementioned device and 25 cm^2^ Tryptone Soya Agar with Disinhibitor contact plates (OXOID Deutschland GmbH, Wesel, Germany) for a total of 72 samples.

The fogging was carried out using a fully automatic Aerosept 500 dry mist aerosol generator (Laboratories ANIOS, Lille-Hellemmes, France). The device was positioned at the start of the vehicle (at the driver’s first door) and the fog nozzles were aimed at the rear of the vehicle. Upon the device initiation, it was necessary to provide the size of the fogging space (height × length × width in meters) to accurately calculate the amount of disinfectant sufficient to decontaminate the room. The ready-to-use liquid Aseptanios AD (Laboratoires ANIOS, Lille-Hellemmes, France) containing peracetic acid stabilized with acetic acid and hydrogen peroxide was used for disinfection. The fogging was carried out immediately after the transportation of passengers and lasted two and a half hours.

After disinfection, all windows and doors were opened to allow prompt evaporation of the excess of the disinfectant. After 30 min of airing, samples for microbiological testing were taken again from the same four surfaces as before. A total of 72 impressions were made.

Subsequently, the contact plates were incubated for 48 h at 35 °C ± 2 °C. After removal from the incubator, the number of colonies on the plate surface was counted and reported in Colony Forming Units per 25 cm^2^ (CFU/25 cm^2^).

### 2.3. Identification of Microorganisms

To obtain separate bacterial cultures, isolation on Columbia Agar with Sheep Blood plates (OXOID Deutschland GmbH, Wesel, Germany) was conducted. Then, the inoculated media were incubated in aerobic conditions for 24 h at 35 °C ± 2 °C.

Microbial identification was performed on a VITEK**^®^** MS mass spectrometer (bioMérieux, Marcyl’Etoile, France) using MALDI-TOF (Matrix Assisted Laser Desorption Ionization Time-of-Flight) technology. A disposable plate with a total of 48 test points was used for the analysis of microorganisms. A single colony of bacteria was deposited on the target slide, and then the CHCA matrix (4-hydroxy-α-cyanocinnamic acid) was added. Accordingly, the prepared plate was air dried, and then placed in the VITEK**^®^** MS instrument. Microbial identification was made by obtaining spectra using MALDI-TOF technology and spectral analysis using the VITEK MS database.

**Figure 2:**
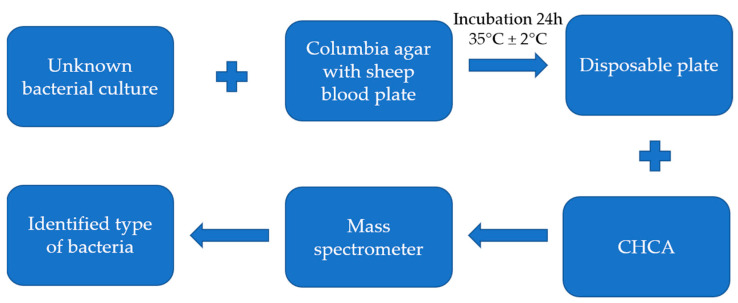
Process of identifying the type of bacteria.

The entire process of identifying the type of bacteria is shown in Figure 2.

## 3. Results

### 3.1. Prevalence of Bacteria on Tested Surfaces

The most prevalent bacteria were *Micrococcus luteus* (*M. luteus*) and *Bacillus licheniformis* (*B. licheniformis*). In coaches, *M. luteus* was present on all surfaces with highest prevalence on windows and seatbacks—33% and 25%, respectively. The percentage of colonies of *M. luteus* on buses’ walls and seatbacks was 14% and 19%, respectively. *Staphylococcus epidermidis* (*S. epidermidis*) was only present in buses, whereas *Staphylococcus hominis* (*S. hominis*) and *Exiguobacterium acetylicum* (*E. acetylicum*) were only present in coaches. Detailed data on percentage of bacterial colonies on individual surfaces are presented in Table 2.

### 3.2. Effectiveness of the Disinfection

**Figure 3:**
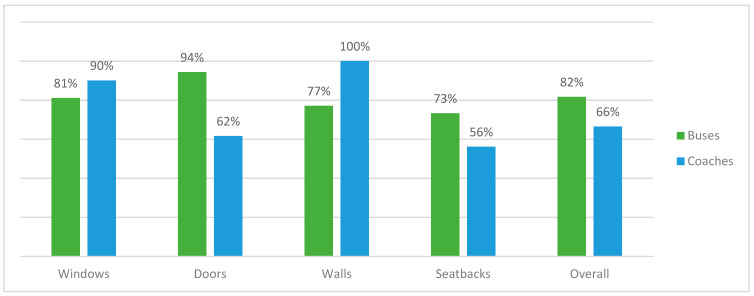
The effectiveness of disinfection on different surfaces.

The study also showed a substantial inactivation effect of microorganisms by disinfection using PAA fumigation. The overall effectiveness of disinfection was 81.7% in buses and 66.5% in coaches (*p* < 0.001 and 0.001 respectively). In buses, the highest percentage reduction was noted on doors (94.3%; *p* = 0.01) and windows (81.2%; *p* = 0.02), while in coaches the highest reduction was reported on windows (90.1%; *p* = 0.01) and walls (100%; *p* = 0.04). The lowest inactivation effect was observed on coaches’ seatbacks and doors—56.1% and 61.6% respectively. Detailed data of the effect of fogging in the number of colonies before and after disinfection are presented in Table 3 and the effectiveness of the disinfection is illustrated in Figure 3.

## 4. Discussion

Public transportation facilitates the spread of potentially pathogenic organisms. This may lead to the transmission of various diseases in the community. In Turkey, the researchers found *Staphylococcus aureus* (*S. aureus*) and coagulase-negative staphylococci (CNS) on the handles of the metrobuses, and additionally they found several species of *Enterococcus* on the handles of the buses [36]. CNS, including *S. epidermidis*, *S. hominis* and *Staphylococcus haemolyticus* (*S. haemolyticus*), were also found in subway stations in Shanghai, China [37]. In accordance with their findings, we also isolated members of the coagulase-negative staphylococci, including *S. epidermis* and *S. hominis*. Another substantial group found both in buses and coaches were *Bacillus strains*, including *B. licheniformis* and *Bacillus cereus* (*B. cereus*).

In Chengdu, China Wu et al. assessed shared bicycles, increasingly chosen instead of public transportation, and found *Bacillus* and *Staphylococcus* colonies to be present in the majority of the tested microbial community [38]. Similar findings, including the presence of *B. cereus*, *Bacillus anthracis* (*B. anthracis*) and *S. aureus*, were reported in subway stations of New York City, USA. The authors noted that only 31% of the investigated microbial community were potentially opportunistic pathogens [39].

Currently, there is no available research regarding the use of dry fog disinfection with peracetic acid in public transport, such as buses or coaches. Richter et al. in 2018 conducted research using this disinfection method in the subway and used materials from a retired metro railcar. They showed that disinfection of PAA and H_2_O_2_ in the subway on different materials did not completely inactivate *B. anthracis* spores. The effectiveness of decontamination largely depended on the material used and reached approximately 41% for *B. anthracis* Ames and 38% for *Bacillus atrophaeus* (*B. atrophaeus*) [40]. In our study the effectiveness differed depending on the material of the sampling site, with highest efficacy of over 90% on buses’ glass doors and coaches’ windows, and lowest effectiveness of 56% in coaches and 73% in buses on seatbacks’ fabric.

The study conducted by Portner and Hoffman proved that peracetic acid in the form of vapor at 25 °C and variable humidity levels (relative humidity (RH) 40–80%) shows good sporicidal activity against *Bacillus subtilis* (*B. subtilis*) var. *niger* spores on glass and paper. The sporicidal rate was negligible at low RH and the highest in 80% humidity. In contrast to the outcome of the research led by Grand et al., we found that glass is easily decontaminated by peracetic acid thus our findings are also consistent with those of Portner and Hoffmann. In general, spores on the impermeable surfaces tend to be more difficult to kill than those on the porous surface, possibly due to the accumulation of cells on the impermeable surface impeding vapor penetration [41,42]. In addition, microorganisms rest on the surface of smooth objects enabling direct contact with the disinfectant. Absorbent materials, such as fabrics and wood, require the fumigant to firstly penetrate the material before getting in contact with microbes, which negatively impact its effectiveness [43,44]. Furthermore, various substances react with disinfectant differently. The research of Horn and Niemeyer showed that copper and brass enhanced decomposition of PAA, reducing the total amount of peracetic acid on oligodynamic surfaces and consequently resulting in decreased effectivity of the disinfection [45].

The method of PAA decontamination with the use of dry fog is widely used in medical centers due to its low toxicity, biodegradability, ease of use and high efficiency [46,47]. Many studies confirmed high effectivity of peracetic acid disinfection in hospital settings. Solution with H_2_O_2_ and PAA proved effective against multidrug-resistant organisms, such as imipenem resistant *Acinetobacter baumannii* (*A. baumannii*), methicillin-resistant *Staphylococcus aureus* (MRSA) and ceftazidime resistant *Pseudomonas aeruginosa* (*P. aeruginosa*) in Intensive Care Units [48]. Lee et al. showed very high efficacy (>99%) of disinfection of endoscopes with peracetic acid [49]. Different study also proved good sterilization effect of PAA on flexible endoscopes on many bacterial species, including MRSA, vacomycin-resitant *Enterococcus* and *Clostridium difficile* (*C. difficile*) [50]. In addition, PAA is used for disinfecting personal protective equipment, even more so during the COVID-19 pandemic. Studies demonstrated good biocidal effect of peracetic acid on SARS-CoV-2 on N95 respirators. Furthermore, decontamination did not cause any changes in filtration efficiency of the masks proving that appropriate decontamination enables re-use of the N95 respirators without posing a risk to the healthcare workers [51,52]. In a different study on the inactivation of the SARS-CoV-2 virus, the decontamination in the air was proven effective even at a low concentration of aerosolized peroxyacetic acid-hydrogen peroxide (aPAA-HP) [53]. Moreover, Cutts et al. demonstrated the effectiveness of disinfection in SARS-CoV-2 contaminated tissue cultures fumigated with PAA [54]. Thus, research proves that PAA dry fog disinfection can reduce the spread of the SARS-CoV-2.

The greatest limitation of fumigation devices is the necessity to leave the premises for the duration of the disinfection due to the irritating effect and possible toxicity of the fumigant. In addition, handling the solution in preparation for the disinfection might pose a health risk for workers if carried out inappropriately. Although, the research conducted in Regional Hospital of Florence, Italy showed that environmental concentrations of PAA in cases of short-time exposure (10 min) during handling the PAA for fogging did not exceed the lowest level (AEGL1; 0.52 mg/m^3^) of the Acute Exposure Guideline Levels established by the United States Environmental Protection Agency in 87% tested units, confirming that it is relatively safe for healthcare workers performing the disinfection. The levels of PAA did not exceed AEGL2 (1.6 mg/m^3^) in any of the units. They noted that exceeded levels of PAA were reported in units with poor ventilation pointing out the importance of evaluating PAA levels in the workplace to maintain workers’ safety [55].

The limitations of our study include the lack of measurement of temperature and humidity level in disinfected vehicles, which could affect the fumigation effectiveness.

## 5. Conclusions

Finally, our research confirmed the effectiveness of the dry fog disinfection method with peracetic acid stabilized with hydrogen peroxide and acetic acid in public transport vehicles. It has been shown that disinfecting bus and coach cabins reduces the number of bacterial colonies by 81.7% and 66.5%, respectively. To summarize, it is an easy-to-use, and portable technology that could be considered for transportation disinfection.

## Figures and Tables

**Table 1:** Characteristics of types of bacteria prevalent in public transport.

Type of Bacteria	Shape of Bacteria	Gram Staining	Occurrence	An Opportunistic Infection	Pathogenic Species
*Staphylococcus epidermidis*	grains	positive	human mucous membranes and skin	nosocomial sepsis	-
*Staphylococcus hominis*	grains	positive	human skin	skin and soft tissue infections	-
*Micrococcus luteus*	spherical	positive/variable	human skin, mouth, mucosae, oropharynx, upper respiratory tract	bacteremia	-
*Exiguobacterium acetylicum*	rod	positive	demographic diversity	bacteremia	-
*Bacillus cereus*	rod	positive	ubiquitous in the environment and food products	pneumonia, infective endocarditis, meningitis, ocular inflammation	food poisoning (diarrhea, vomiting)
*Bacillus licheniformis*	rod	positive	in the soil; on the skin and feathers of birds	ventriculitis, ophthalmitis, bacteremia, peritonitis, endocarditis	-

**Table 2:** Percentage of bacterial colonies on individual surfaces.

	Buses		Coaches	
	Name of the Bacterial Colony	%	Name of the Bacterial Colony	%
Windows	*Staphylococcus epidermidis*	15.4	*Micrococcus luteus*	33.3
	*Bacillus licheniformis*	15.4		
Doors	*Bacillus* *cereus*	14.3	*Bacillus licheniformis*	11.5
			*Micrococcus luteus*	11.5
Walls	*Micrococcus luteus*	14.6	*Micrococcus luteus*	12.2
			*Bacillus licheniformis*	9.8
			*Bacillus* *cereus*	9.8
			*Staphylococcus hominis*	9.8
Seatbacks	*Micrococcus luteus*	19.0	*Micrococcus luteus*	25.0
	*Staphylococcus epidermidis*	14.3	*Staphylococcus hominis*	15.0
			*Exiguobacterium acetylicum*	15.0

**Table 3:** Disinfection effect on various surfaces in buses and coaches.

Buses					Coaches			
Mean Number of Colonies [CFU/25 cm^2^]					Mean Number of Colonies [CFU/25 cm^2^]			
	Before Disinfection	After Disinfection	Reduction [%]	*p*-Value	Before Disinfection	After Disinfection	Reduction [%]	*p*-Value
Windows	2.33	0.44	81.16	0.02	2.22	0.22	90.09	0.01
Doors	3.89	0.22	94.34	0.01	5.78	2.22	61.59	0.01
Walls	1.44	0.33	77.08	0.04	0.67	0	100	0.04
Seatbacks	4.56	1.22	73.25	0.01	4.56	2	56.14	0.02
Overall	3.06	0.56	81.70	<0.001	3.31	1.11	66.47	0.001

## Data Availability

The research data included in the analysis are the authors’ content. The datasets used and/or analyzed during the present study are available from the corresponding author on reasonable request.

## References

[1] Das P., Upadhyay R.K., Misra A.K., Rihan F.A., Das P., Ghosh D. (2021). Mathematical model of COVID-19 with comorbidity and controlling using non-pharmaceutical interventions and vaccination. Nonlinear Dyn..

[2] Das P., Nadim S.S., Das S., Das P. (2021). Dynamics of COVID-19 transmission with comorbidity: A data driven modelling based approach. Nonlinear Dyn..

[3] Chang A., Schnall A.H., Law R., Bronstein A.C., Marraffa J.M., Spiller H.A., Hays H.L., Funk A.R., Mercurio-Zappala M., Calello D.P. (2020). Cleaning and disinfectant chemical exposures and temporal associations with COVID-19—National Poison Data System, United States, 1 January 2020–31 March 2020. Morb. Mortal. Wkly. Rep..

[4] Gharpure R., Hunter C.M., Schnall A.H., Barrett C.E., Kirby A.E., Kunz J., Berling K., Mercante J.W., Murphy J.L., Garcia-Williams A.G. (2020). Knowledge and Practices Regarding Safe Household Cleaning and Disinfection for COVID-19 Prevention—United States, May 2020. Morb. Mortal. Wkly. Rep..

[5] Fraise A. (2011). Currently available sporicides for use in healthcare, and their limitations. J. Hosp. Infect..

[6] Rutala W.A., Weber D.J. (2013). Disinfectants used for environmental disinfection and new room decontamination technology. Am. J. Infect. Control.

[7] Havill N.L. (2013). Best practices in disinfection of noncritical surfaces in the health care setting: Creating a bundle for success. Am. J. Infect. Control.

[8] Shen J., Duan H., Zhang B., Wang J., Ji J.S., Wang J., Pan L., Wang X., Zhao K., Ying B. (2020). Prevention and control of COVID-19 in public transportation: Experience from China. Environ. Pollut..

[9] Kleinschmidt S., Huygens F., Faoagali J., Rathnayake I.U., Hafner L.M. (2015). *Staphylococcus epidermidis* as a cause of bacteremia. Future Microbiol..

[10] Qin L., Da F., Fisher E.L., Tan D.C.S., Nguyen T.H., Fu C.L., Tan V.Y., McCausland J.W., Sturdevant D.E., Joo H.S. (2017). Toxin mediates sepsis caused by methicillin-resistant *Staphylococcus epidermidis*. PLoS Pathog..

[11] Rogers K.L., Fey P.D., Rupp M.E. (2009). Coagulase-negative staphylococcal infections. Infect. Dis. Clin. N. Am..

[12] Natsis N.E., Cohen P.R. (2018). Coagulase-Negative Staphylococcus Skin and Soft Tissue Infections. Am. J. Clin. Dermatol..

[13] Szczuka E., Krzymińska S., Bogucka N., Kaznowski A. (2018). Multifactorial mechanisms of the pathogenesis of methicillin-resistant *Staphylococcus hominis* isolated from bloodstream infections. Antonie Van Leeuwenhoek.

[14] Zhu M., Zhu Q., Yang Z., Liang Z. (2021). Clinical Characteristics of Patients with *Micrococcus luteus* Bloodstream Infection in a Chinese Tertiary-Care Hospital. Pol. J. Microbiol..

[15] Martín Guerra J.M., Martín Asenjo M., Rodríguez Martín C. (2019). Bacteraemia by *Micrococcus luteus* in an inmunocompromised patient. Med. Clin..

[16] Chauhan H., Bagyaraj D.J., Selvakumar G., Sundaram S.P. (2015). Novel plant growth promoting rhizobacteria—Prospects and potential. Appl. Soil Ecol..

[17] Jessberger N., Dietrich R., Granum P.E., Märtlbauer E. (2020). The *Bacillus cereus* Food Infection as Multifactorial Process. Toxins.

[18] Bottone E.J. (2010). *Bacillus cereus*, a volatile human pathogen. Clin. Microbiol. Rev..

[19] Salkinoja-Salonen M.S., Vuorio R., Andersson M.A., Kämpfer P., Andersson M.C., Honkanen-Buzalski T., Scoging A.C. (1999). Toxigenic strains of *Bacillus licheniformis* related to food poisoning. Appl. Environ. Microbiol..

[20] Haimi S., Vienonen A., Hirn M., Pelto M., Virtanen V., Suuronen R. (2008). The effect of chemical cleansing procedures combined with peracetic acid–ethanol sterilization on biomechanical properties of cortical bone. Biologicals.

[21] Humphreys P.N., Finan P., Rout S., Hewitt J., Thistlethwaite P., Barnes S., Pilling S.A. (2013). systematic evaluation of a peracetic-acid-based high performance disinfectant. J. Infect. Prev..

[22] Otterspoor S., Farrell J. (2019). An evaluation of buffered peracetic acid as an alternative to chlorine and hydrogen peroxide based disinfectants. Infect. Dis. Health.

[23] Sisti M., Brandi G., Santi M.D., Rinaldi L., Schiavano G.F. (2012). Disinfection efficacy of chlorine and peracetic acid alone or in combination against *Aspergillus* spp. and *Candida albicans* in drinking water. J. Water Health.

[24] Ríos-Castillo A.G., González-Rivas F., Rodríguez-Jerez J.J. (2017). Bactericidal efficacy of hydrogen peroxide-based disinfectants against Gram-positive and Gram-negative bacteria on stainless steel surfaces. J. Food Sci..

[25] Humayun T., Qureshi A., Roweily S.F.A., Carig J., Humayun F. (2019). Efficacy of hydrogen peroxide fumigation in improving disinfection of hospital rooms and reducing the number of microorganisms. J. Ayub Med. Coll. Abbottabad.

[26] String G.M., Gutiérrez E.V., Lantagne D.S. (2020). Laboratory efficacy of surface disinfection using chlorine against Vibrio cholerae. J. Water Health.

[27] Ning P., Shan D., Hong E., Liu L., Zhu Y., Cui R., Zhou Y., Wang B. (2020). Disinfection performance of chlorine dioxide gas at ultra-low concentrations and the decay rules under different environmental factors. J. Air Waste Manag. Assoc..

[28] Donskey C.J. (2013). Does improving surface cleaning and disinfection reduce health care-associated infections?. Am. J. Infect. Control.

[29] Burfoot D., Hall K., Brown K., Xu Y. (1999). Fogging for the disinfection of food processing factories and equipment. Trends Food Sci. Technol..

[30] Leggett M.J., Schwarz J.S., Burke P.A., McDonnell G., Denyer S.P., Maillard J.Y. (2015). Mechanism of Sporicidal Activity for the Synergistic Combination of Peracetic Acid and Hydrogen Peroxide. Appl. Environ. Microbiol..

[31] Costa A., Colosio C., Gusmara C., Sala V., Guarino M. (2014). Effects of disinfectant fogging procedure on dust, ammonia concentration, aerobic bacteria and fungal spores in a farrowing-weaning room. Ann. Agric. Environ. Med..

[32] Barbut F., Menuet D., Verachten M., Girou E. (2009). Comparison of the efficacy of a hydrogen peroxide dry-mist disinfection system and sodium hypochlorite solution for eradication of *Clostridium difficile* spores. Infect. Control Hosp. Epidemiol..

[33] Deshpande A., Mana T.S., Cadnum J.L., Jencson A.C., Sitzlar B., Fertelli D., Hurless K., Kundrapu S., Sunkesula V.C., Donskey C.J. (2014). Evaluation of a sporicidal peracetic acid/hydrogen peroxide-based daily disinfectant cleaner. Infect. Control Hosp. Epidemiol..

[34] Rybka A., Gavel A., Kroupa T., Meloun J., Prazak P., Draessler J., Pavlis O., Kubickova P., Kratzerova L., Pejchal J. (2021). Peracetic acid-based disinfectant is the most appropriate solution for a biological decontamination procedure of responders and healthcare workers in the field environment. J. Appl. Microbiol..

[35] Kruszewska E., Grześ H., Czupryna P., Pancewicz S., Groth M., Wondim M., Moniuszko-Malinowska A. (2021). Fogging With Peracetic Acid in Schools and Kindergartens. Front. Public Health.

[36] Birteksöz Tan A.S., Erdoğdu G. (2017). Microbiological burden of public transport vehicles. Istanbul. J. Pharm..

[37] Zhou F., Wang Y. (2013). Characteristics of antibiotic resistance of airborne *Staphylococcus* isolated from metro stations. Int. J. Environ. Res. Public Health.

[38] Wu Y., Xie J., Li J., Zhao J., Qiao S., Li Y., Zeng J. (2021). Shared bicycle microbial community: A potential antibiotic-resistant bacteria warehouse. Folia Microbiol..

[39] Afshinnekoo E., Meydan C., Chowdhury S., Jaroudi D., Boyer C., Bernstein N., Maritz J.M., Reeves D., Gandara J., Chhangawala S. (2015). Geospatial Resolution of Human and Bacterial Diversity with City-Scale Metagenomics. Cell Syst..

[40] Richter W.R., Wood J.P., Wendling M.Q.S., Rogers J.V. (2018). Inactivation of *Bacillus anthracis* spores to decontaminate subway railcar and related materials via the fogging of peracetic acid and hydrogen peroxide sporicidal liquids. J. Environ. Manag..

[41] Portner D.M., Hoffman R.K. (1968). Sporicidal effect of peracetic acid vapor. Appl. Microbiol..

[42] Grand I., Bellon-Fontaine M.N., Herry J.M., Hilaire D., Moriconi F.X., Naïtali M. (2010). The resistance of *Bacillus atrophaeus* spores to the bactericidal activity of peracetic acid is influenced by both the nature of the solid substrates and the mode of contamination. J. Appl. Microbiol..

[43] Rogers J.V., Richter W.R., Shaw M.Q., Shesky A.M. (2009). Large-Scale Inactivation of *Bacillus anthracis* Ames, Vollum, and Sterne Spores Using Vaporous Hydrogen Peroxide. Appl. Biosaf..

[44] Rogers J.V., Sabourin C.L., Choi Y.W., Richter W.R., Rudnicki D.C., Riggs K.B., Taylor M.L., Chang J. (2005). Decontamination assessment of *Bacillus anthracis*, *Bacillus subtilis*, and *Geobacillus stearothermophilus* spores on indoor surfaces using a hydrogen peroxide gas generator. J. Appl. Microbiol..

[45] Horn H., Niemeyer B. (2020). Aerosol disinfection of bacterial spores by peracetic acid on antibacterial surfaces and other technical materials. Am. J. Infect. Control.

[46] Gregersen J.P., Roth B. (2012). Inactivation of stable viruses in cell culture facilities by peracetic acid fogging. Biologicals.

[47] Mana T.S., Sitzlar B., Cadnum J.L., Jencson A.L., Koganti S., Donskey C.J. (2017). Evaluation of an automated room decontamination device using aerosolized peracetic acid. Am. J. Infect. Control.

[48] Blazejewski C., Wallet F., Rouzé A., Le Guern R., Ponthieux S., Salleron J., Nseir S. (2015). Efficiency of hydrogen peroxide in improving disinfection of ICU rooms. Crit. Care.

[49] Lee J.M., Lee K.M., Kim D.B., Go S.E., Ko S., Kang Y., Hong S. (2018). Efficacy of Peracetic Acid (EndoPA^®^) for Disinfection of Endoscopes. Korean J. Gastroenterol..

[50] Chenjiao W., Hongyan Z., Qing G., Xiaoqi Z., Liying G., Ying F. (2016). In-Use Evaluation of Peracetic Acid for High-Level Disinfection of Endoscopes. Gastroenterol. Nurs..

[51] Kumar A., Kasloff S.B., Leung A., Cutts T., Strong J.E., Hills K., Gu F.X., Chen P., Vazquez-Grande G., Rush B. (2020). Decontamination of N95 masks for re-use employing 7 widely available sterilization methods. PLoS ONE.

[52] Cadnum J.L., Li D.F., Redmond S.N., John A.R., Pearlmutter B., Donskey C.J. (2020). Effectiveness of Ultraviolet-C Light and a High-Level Disinfection Cabinet for Decontamination of N95 Respirators. Pathog. Immun..

[53] Schinköthe J., Scheinemann H.A., Diederich S., Freese H., Eschbaumer M., Teifke J.P., Reiche S. (2021). Airborne Disinfection by Dry Fogging Efficiently Inactivates Severe Acute Respiratory Syndrome Coronavirus 2 (SARS-CoV-2), Mycobacteria, and Bacterial Spores and Shows Limitations of Commercial Spore Carriers. Appl. Environ. Microbiol..

[54] Cutts T., Kasloff S., Safronetz D., Krishnan J. (2021). Decontamination of common healthcare facility surfaces contaminated with SARS-CoV-2 using peracetic acid dry fogging. J. Hosp. Infect..

[55] Pacenti M., Dugheri S., Boccalon P., Arcangeli G., Dolara P., Cupelli V. (2010). Air monitoring and assessment of occupational exposure to peracetic acid in a hospital environment. Ind. Health.

